# Effects of Neuropeptide Y on Stem Cells and Their Potential Applications in Disease Therapy

**DOI:** 10.1155/2017/6823917

**Published:** 2017-10-03

**Authors:** Song Peng, You-li Zhou, Zhi-yuan Song, Shu Lin

**Affiliations:** ^1^Department of Cardiology, Southwest Hospital, Third Military Medical University, Chongqing, China; ^2^School of Medicine, University of Wollongong and Illawarra Health and Medical Research Institute, Wollongong, NSW 2522, Australia

## Abstract

Neuropeptide Y (NPY), a 36-amino acid peptide, is widely distributed in the central and peripheral nervous systems and other peripheral tissues. It takes part in regulating various biological processes including food intake, circadian rhythm, energy metabolism, and neuroendocrine secretion. Increasing evidence indicates that NPY exerts multiple regulatory effects on stem cells. As a kind of primitive and undifferentiated cells, stem cells have the therapeutic potential to replace damaged cells, secret paracrine molecules, promote angiogenesis, and modulate immunity. Stem cell-based therapy has been demonstrated effective and considered as one of the most promising treatments for specific diseases. However, several limitations still hamper its application, such as poor survival and low differentiation and integration rates of transplanted stem cells. The regulatory effects of NPY on stem cell survival, proliferation, and differentiation may be helpful to overcome these limitations and facilitate the application of stem cell-based therapy. In this review, we summarized the regulatory effects of NPY on stem cells and discussed their potential applications in disease therapy.

## 1. Introduction

NPY, a 36-amino acid neuropeptide, was first isolated by Tatemoto et al. from swine brain in 1982 [1]; it belongs to the pancreatic polypeptide family together with pancreatic polypeptide (PP) and peptide YY (PYY). NPY, whose structure is characterized by a large number of tyrosine residues (5 of 36 amino acid residues) and an amidated C-terminal group, remained highly conserved among species in the course of evolution [2]. As one of the most abundant neuropeptides, NPY is widely present in the central and peripheral nervous systems (CNS/PNS) and is a crucial mediator for other peripheral tissues. In the CNS, it is distributed in regions such as the cerebral cortex, hypothalamus, brainstem, hippocampus, striatum, and limbic structures [2–4]. In the PNS, it is expressed in sympathetic ganglia and costored and coreleased with noradrenaline during sympathetic nerve stimulation [5]. Mounting evidence indicates that NPY expresses in many peripheral tissues such as the retina, bone, adipose tissue, adrenal medulla, and platelets [6–10]. Consistent with its wide distribution, NPY has been implicated in a variety of biological processes including food intake, circadian rhythm, energy metabolism, cardiovascular function, and neuroendocrine secretion [11–15].

Five NPY receptors (Y1, Y2, Y4, Y5, and y6) have been identified in mammals, which all belong to the super family of G protein-coupled receptors. However, the Y4 receptor has limited affinity for NPY [16]. The y6 receptor is not functional in primates as their y6 gene exists in a truncated version missing the seventh transmembrane domain [17, 18]. NPY receptors are also widely distributed in central and peripheral tissues of which each receptor exhibits different distributions and mediates their specific functions [19].

Stem cells are a kind of primitive and undifferentiated cells which are characterized by perpetual self-renewal and the potency to differentiate into specialized cell types. Based on their origin, stem cells can be categorized into two types: embryonic stem cells (ESCs) and non-ESCs [20]. The non-ESCs are derived from adult and fetal tissues including hematopoietic stem cells, bone marrow mesenchymal stem cells, adipose-derived stem cells, neural stem cells, and dental pulp stem cells [21]. Stem cells have the therapeutic potential to replace damaged cells, secrete paracrine molecules, promote angiogenesis, modulate immunity, and facilitate tissue repair [22, 23]. Hence, the efficacy of stem cell-based therapy has been described in many diseases including myocardial infarction, stroke, neuritis, liver cirrhosis, pulmonary fibrosis, spinal cord injuries (SCI), Parkinson's disease, and Alzheimer's disease [24–31]. However, some limitations still hamper the application of stem cell-based therapy, such as poor survival, oncogenic potential, and low differentiation and integration rates, which need to be further researched to open up new avenues for the therapy.

## 2. Effects of NPY on Stem Cells

**Figure 1:**
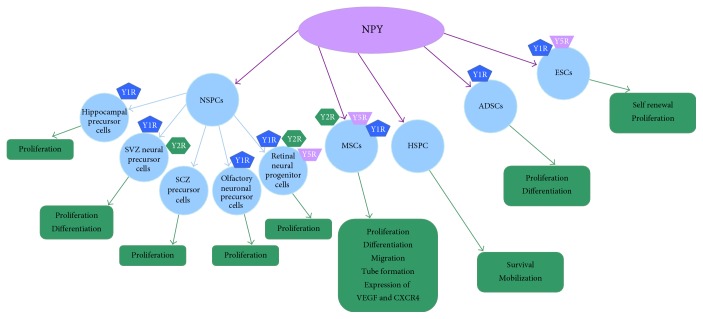
Main effects of NPY on different stem cells. NPY exerts multiple regulatory effects on MSC functions, including proliferation (via Y5R), differentiation (via Y2R and Y1R), migration, tube formation, and expression of VEGF and CXCR4. NPY could promote the proliferation and differentiation of NSPCs through corresponding Y receptors. Meanwhile, NPY could stimulate the proliferation and differentiation of ADSCs as well as the survival and mobilization of HSPC. In addition, exogenous NPY supports the long-term self-renewal and proliferation of undifferentiated ESCs via the Y1 and Y5 receptors. In the figure, the NPY receptor symbols symbolized their functional involvement in the regulation of NPY on different stem cells. NSPCs—neural stem/precursor cells; MSCs—mesenchymal stem cells; HSPC—hematopoietic stem/progenitor cells; ADSCs—adipose-derived stem cells; ESCs—embryonic stem cells; SVZ—subventricular zone; SCZ—subcallosal zone.

Increasing researches indicate that NPY exerts regulatory effects on the proliferation, differentiation, and survival of stem cells, which is speculated to have potential applications in treatment for many diseases. Here, we reviewed the effects of NPY on different stem cells and the involved mechanisms (Figure 1).

### 2.1. NPY and Neural Stem/Precursor Cells (NSPCs)

#### 2.1.1. Hippocampal Precursor Cells

Howell et al. uncovered that NPY increased the neurosphere formation of early postnatal rat-derived primary hippocampal cultures as well as the 5-bromo-2-deoxyuridine (BrdU) incorporation of nestin^+^ hippocampal precursor cells, which indicated that NPY could promote the proliferation of hippocampal precursor cells. Besides using NPY receptor agonists and antagonists as well as Y1 receptor knockout (Y1^−/−^) mice, they further demonstrated that the proliferative effect of NPY on hippocampal precursor cells was mediated via Y1 receptor [32]. Subsequent study of this research group proved that NPY displayed proliferative effect on neuronal precursors in the dentate gyrus. There were increased total cells, neurons (class III *β*-tubulin expressing cells), and cell proliferation in postnatal rat-derived dentate gyrus cells cultured in medium with l *μ*M NPY than that cultured in control medium, of which the neuroproliferative effect of NPY was mediated by Y1 receptor and involved ERK1/2 activation. Meanwhile, in Y1^−/−^ mice, the proliferation rate and immature doublecortin-positive neurons in the dentate gyrus were significantly reduced [33]. In vivo, exogenous NPY also exhibits proliferative effect on neural progenitors in the dentate gyrus. Decressac et al. demonstrated that intracerebroventricular (ICV) injection of NPY to C57BL/6 mice promoted the proliferation of neural progenitors and neuroblasts in the dentate gyrus, and ICV injection of Y1 receptor agonist could mimic this proliferative effect. However, when coinjected with Y1 receptor antagonist or injected to Y1^−/−^ mice, NPY could not induce the proliferation in the dentate gyrus, indicating that the proliferative effect of NPY is mediated via Y1 receptor [34]. In short, the mechanism involved in NPY-stimulated hippocampal precursor cell proliferation may be mediated by Y1 receptor/intracellular nitric oxide (NO)/cyclic guanosine monophosphate (cGMP)/cGMP-dependent protein kinase (PKG)/extracellular signal-regulated kinase (ERK) 1/2 signaling pathway [35].

In the aspect of promoting hippocampal precursor proliferation, NPY is able to display synergistic action with other proliferative factors. Rodrigo and colleagues found that the combination of NPY and fibroblast growth factor 2 (FGF2) more potently stimulated proliferation of nestin^+^ hippocampal neural stem progenitor cells (NSPCs) than either factor alone. On the one hand, NPY increased FGF receptor1 (FGFR1) mRNA and protein expression in NSPCs. On the other hand, NPY augmented the proliferative effect of FGF2 via Y1 receptor through shortening the cell cycle time of NSPCs [36].

The proliferative effect of NPY on hippocampal precursor cells presents not only in normal condition but also in pathological conditions. NPY^−/−^ mice displayed lower level of BrdU incorporation in dentate gyrus subgranular zone (SGZ) under the conditions of basal and kainic acid- (KA-) induced seizure, which suggested a role for NPY in seizure-induced proliferation of hippocampal precursor cells [37]. Baptista et al. administered the psychostimulant drug methamphetamine (METH) to dentate gyrus-derived neurosphere cultures and found METH decreased neuronal differentiation of the neurosphere cultures at low concentration and induced cell death at high concentration. Besides, NPY prevented METH-reduced neuronal differentiation and METH-induced cell death, meanwhile NPY promoted the proliferation of dentate gyrus-derived neurosphere cultures [38].

#### 2.1.2. Subventricular Zone (SVZ) Neural Precursor Cells

Agasse et al. gave mice-derived SVZ neurosphere NPY treatment and found that NPY promoted the proliferation and neuronal differentiation of SVZ cells and, particularly, the proneurogenic effect of NPY was mediated via Y1 receptor and involved activation of ERK1/2 and stress-activated protein kinase (SAPK)/c-Jun-NH2-terminal kinase (JNK) signaling [39]. The regulatory effects of NPY on SVZ neural precursors were further demonstrated in vivo. Compared with wild-type (WT) mice, there were significantly reduced proliferating precursor cells and neuroblasts in SVZ of Y1^−/−^ and Y2^−/−^ mice [40]. Researchers administrated ICV injection of saline, NPY, Y1 receptor agonist, or a mixed solution of Y1 receptor antagonist and NPY, respectively, to adult mice and investigated the proliferation of SVZ cells at 48 h after injection. The results showed that NPY and Y1 receptor agonist stimulated the proliferation of SVZ neural precursors significantly. In contrast, the proliferative effect of NPY was inhibited by Y1 receptor antagonist, which suggested Y1 receptor mediated this proliferative effect of NPY. Meanwhile, the researchers substantiated that NPY augmented the migration of newly generated neuroblasts which were formed in the SVZ toward the striatum and olfactory bulb through rostral migratory stream [41]. Additionally, Thiriet et al. discovered that SVZ cells produced endogenous NPY which indicated that NPY may act as a paracrine/autocrine factor in SVZ. They also demonstrated that NPY promoted the proliferation and neuronal differentiation of SVZ cells, which were isolated from newborn rats, via Y1 receptor-mediated ERK1/2 MAP kinase pathway [42]. Accordingly, these results indicated that NPY could promote the proliferation and neuronal differentiation of SVZ neural precursor cells via Y1 receptor; although Y2^−/−^ mice also had reduced proliferating precursor cells and neuroblasts in SVZ as Y1^−/−^ mice, the exact effects of Y2 receptor in proliferation and differentiation of SVZ neural precursor cells were still unclear which may be mediated by Y2 receptor itself or by regulating Y1 receptor expression or by other mechanisms. Further studies are needed to better understand this process.

#### 2.1.3. Subcallosal Zone (SCZ) Precursor Cells

Unlike the hippocampus and subventricular zone around the lateral ventricle which regenerate neurons in mammalian adulthood, SCZ predominately generates oligodendrocytes. Interestingly, seizure could promote the SCZ activity thereby producing more glial progenitors which migrate to the impaired hippocampus [43]. It has been reported that NPY^−/−^ mice had significantly reduced proliferative (BrdU positive) cells in the left subcallosal zone under basal condition; meanwhile, seizure-induced proliferation in the SCZ of NPY^−/−^ mice also decreased compared with WT control, which suggested that NPY played a role in the proliferation of SCZ precursor cells [44]. Thus, it points out a significative possibility that NPY promotes hippocampal neurogenesis through inducing SCZ oligodendrogliogenesis.

#### 2.1.4. Olfactory Neuronal Precursor Cells

The neuroproliferative effect of NPY was primarily identified in olfactory epithelium. Hansel and colleagues uncovered that NPY^−/−^ mice had decreased proliferation of olfactory neuronal precursor cells in the olfactory epithelium. They performed experiments with primary olfactory cultures to demonstrate that NPY facilitated the proliferation of neuronal precursor cells via Y1 receptor and involved protein kinase C (PKC)/ERK1/2 signaling [45]. The role of NPY and Y1 receptor in proliferation of olfactory precursor cells was further confirmed in subsequent studies. Doyle et al. reported that primary adult olfactory precursor cells derived from NPY^−/−^, NPY/PYY^−/−^, and Y1^−/−^ mice formed reduced olfactory neurospheres compared with WT mice and only WT control-derived glandular cells could form secondary neurospheres, suggesting an important role of NPY signaling in proliferation of olfactory precursor cells. Furthermore, they also discovered that Y1^−/−^ mice exhibited impaired olfaction compared with WT mice which may result from reduced formation of olfactory neurons [46]. Pathological examination of the olfactory neuroepithelium derived from NPY^−/−^ and WT mice revealed that there were significantly fewer number of olfactory receptor neurons in olfactory neuroepithelium of NPY^−/−^ mice [47].

Furthermore, NPY is associated with neuroregeneration of olfactory epithelium under pathological conditions. In the olfactory epithelium, adenosine triphosphate (ATP) is released upon injury and stimulate proliferation and differentiation of olfactory neuronal precursors via P2 purinergic receptor [48]. It has been reported that intranasal instillation of ATP enhances the expression of NPY in olfactory epithelium sustentacular and microvillous cells via P2 purinergic receptors and NPY stimulates the proliferation of neuronal progenitor cells through Y1 receptor-mediated ERK1/2 activation [49, 50]. A subtype of microvillous cells which express inositol triphosphate receptor subtype 3(IP3R3) plays a critical role in neuroregeneration and recovery after olfactory epithelium injury as they secrete sufficient NPY [51]. A recent study showed that the number of IP3R3^+^NPY^+^ microvillous cells and expression of NPY in mouse olfactory epithelium existed an age-dependent decline. In addition, the number and proliferation of olfactory stem cells also decreased with age, suggesting a role of NPY in age-related olfactory dysfunction [52].

#### 2.1.5. Retinal Neural Progenitor Cells

It has been reported that NPY could promote the proliferation of retinal neural progenitor cells and retinal neural cells. The application of exogenous NPY to primary rat retinal cell cultures for 48 h promoted retinal neural cell proliferation significantly, and the proliferation of nestin^+^ retinal neural progenitor cells in this culture was increased at the same time. These proliferative effects of NPY are mediated via Y1, Y2, and Y5 receptors through activation of NO-cGMP and ERK1/2 pathway [53]. The general opinion is that the mature mammalian retina was short of regenerative capacity [54] but the proliferative effects of NPY on retinal neural progenitor cells and retinal neural cells provide experimental evidence and novel strategy for regeneration of the injured retina.

### 2.2. NPY and Mesenchymal Stem Cells (MSCs)

Wang and colleagues found that pretreating rat bone marrow mesenchymal stem cells (BMSCs) with NPY for 72 h upregulated the expression of vascular endothelial growth factor (VEGF) and genes required for mitosis, including aurora B kinase, FGF-2, cyclin A2, eukaryotic initiation factor 4E, and stromal cell-derived factor-1*α*, when under hypoxia condition. Meanwhile, NPY pretreatment promoted the migration of BMSCs through upregulating CXCR4 expression and induced endothelium differentiation as well as tube formation of BMSCs [55]. It has been reported that NPY enhanced the proliferation of rat bone marrow stromal cells (BMCs) which was mediated via Y5 receptor. The proliferative response to NPY of BMCs isolated from old rats is weaker than those isolated from neonatal and young rats, because of the decreased expression of Y5 receptors in old BMCs. Furthermore, Y5 receptor gene transfection restored the impaired growth potential of old BMCs [56].

Recent studies have demonstrated that NPY system displays regulatory effects on osteoblastic and adipogenic differentiation of MSCs. Compared with wild-type control, BMCs derived from Y2^−/−^ mice cultured under osteogenic and adipogenic conditions exhibited increased mineralization and adipocyte formation, respectively [57]. The Y1 receptor mRNA expression of BMCs cultured from Y2^−/−^ mice was downregulated, which maybe a result from the lack of Y2 receptor signaling relieved the feedback inhibition on NPY release, and elevated NPY caused overstimulation of Y1 receptors and followed by desensitization and downregulation of Y1 receptor [57]. In a follow-up study, Lee et al. reported that BMCs from Y1^−/−^ mice formed more mineralized nodules under osteogenic conditions and more adipocytes under adipogenic conditions, compared with wild-type control. Meanwhile, the MSCs and mesenchymal osteoprogenitor cells isolated from the bone of Y1^−/−^ mice have enhanced ability to differentiate into osteoblasts and adipocytes, which indicated that NPY could suppress the differentiation of mesenchymal progenitor cells via Y1 receptor [58]. Besides, previous research showed that exogenous NPY promoted osteoblast differentiation of primary mouse BMCs through Y2 receptor [59]. However, Liu and colleagues discovered that NPY enhanced osteoblastic differentiation of rat BMSCs in a concentration-dependent manner via Y1 receptor and Wnt signaling pathway [60]. Recent study indicated that NPY promoted the osteogenic differentiation of health volunteer-derived BMSCs and increased the expression of Y1 receptor in human BMSCs [61]. In view of these serial researches, we concluded that in mice, NPY could promote the osteoblastic differentiation of MSCs via Y2 receptor and inhibit MSCs differentiating into osteoblasts via Y1 receptor. Moreover, Y2 receptor exhibited dual feedback inhibition on MSC osteoblastic differentiation through feedback inhibition on NPY release, thereby regulating the expression level of Y1 receptor. However, in rats and human, it has been reported that NPY promoted the osteoblastic differentiation of MSCs through Y1 receptor. These conflicting results in different species need to be further investigated.

### 2.3. NPY and Hematopoietic Stem/Progenitor Cells (HSPC)

It was reported that NPY was of great importance in the survival and mobilization of HSPC. Park et al. uncovered that there were reduced hematopoietic stem cells (HSCs) in the bone marrow of NPY^−/−^ mice and these mice had fewer SNS fibers and CD31^+^ ECs in bone marrow. Through investigating the cause and effect relationships, they concluded that NPY deficiency caused p53-dependent apoptosis of SNS fibers and/or CD31^+^ ECs in the bone marrow, resulting in impairment of HSC survival. Further pharmacological experiments of NPY increase and Y1 receptor intervention illustrated that NPY/Y1 receptor regulation affected HSC survival by reducing apoptosis of SNS fibers and ECs in marrow microenvironment [62]. As a recent study showed, compared with WT control, the HSPC mobilization in NPY^−/−^ mice was impaired which resulted from the decreased matrix metalloproteinase-9 (MMP-9) activity in bone marrow-induced upregulation of HSPC maintenance factors. Besides, exogenous NPY or elevation of endogenous NPY by stress stimulated MMP-9 activity of osteoblast via Y1 receptor thereby suppressing HSPC maintenance factors and inducing HSPC mobilization in mice [63]. Hence, these stimulatory effects of NPY on survival and mobilization of HSPC were mediated through regulating hematopoietic stem cell niche. The HSC niche is critical to support HSC function and to maintain a proper balance between self-renewal and multilineage differentiation. Various types of cells have been identified as HSC niche components which include osteoblasts, stromal cells, vascular endothelial cells, macrophages, adipocytes, and megakaryocytes [64, 65]. Not only osteoblasts that was mentioned above but also vascular endothelial cells, macrophages, and adipocytes have been identified to be regulated by NPY [62, 66, 67], which indicated the potential that NPY could regulate the function of HSPC via these HSC niche component cells. Additionally, previous studies showed that NPY is expressed by bone marrow hematopoietic cells [62, 68] which indicated that HSPC may exert feedback regulation on HSC niche via releasing NPY.

### 2.4. NPY and Adipose-Derived Stem Cells (ADSCs)

Wu et al. researched the effects of NPY at different concentrations on proliferation and differentiation of cultured human adipose-derived stem cells (hADSCs). The results revealed that NPY stimulated the proliferation of hADSCs at low concentrations (10^−14^–10^−11^ mol/L), however, at high concentrations (10^−10^–10^−6^ mol/L), NPY inhibited the proliferation and promoted adipogenic differentiation of hADSCs [69]. Yang et al. uncovered that rat primary preadipocytes and 3T3-L1 cells (a murine preadipocyte cell line) expressed abundant Y1 receptors, and at the same time, NPY promoted the proliferation of rat primary preadipocytes and 3T3-L1 cells in a concentration-dependent manner via Y1 receptor and related to the ERK1/2 pathway [70]. Meanwhile, it was reported that visceral adipose tissue [70] and abdominal subcutaneous adipose tissue [71] synthesized NPY, which indicated that adipose tissue may release NPY in an autocrine manner thereby promoting the proliferation and adipogenic differentiation of its resident ADSCs and adipocyte precursor cells, and consequently result in the further accumulation of the adipose tissue.

### 2.5. NPY and Embryonic Stem Cells (ESCs)

Son and colleagues uncovered that undifferentiated human ESCs expressed NPY, Y1, and Y5 receptors and exogenous NPY supported the long-term self-renewal and proliferation of undifferentiated human ESCs via the Y1 and Y5 receptors involving the activation of AKT, ERK1/2, and cAMP-response element-binding (CREB) signaling [72]. Based on the regulatory effect of NPY on human ESC self-renewal, this group designed a novel chemically defined medium with NPY supplement which could support the long-term culture of human ESCs without feeder cells and serum [72]. The traditional ESC culture needs the support of inactivated mouse embryo fibroblast and fetal calf serum. However, the indeterminacy of feeder cell secretion and potential pathogen contamination of serum hinder the research and application of ESCs. The development of a xeno-free and serum-free medium that supports the long-term culture derivation and large-scale propagation is still one of the major challenges for the clinical application of ESCs. Therefore, the chemically defined medium with NPY supplement may become a novel strategy for ESC translational research. Additionally, NPY takes part in stress-induced adipogenesis of ESCs. As a study showed, stress hormone epinephrine (EPI) facilitated the expression of NPY, Y1, and Y2 receptors in murine ESCs, of which EPI enhanced the adipogenic differentiation of murine ESCs via the NPY system [73].

## 3. Potential Application of NPY's Regulatory Effects on Stem Cells

### 3.1. Neurodegenerative Diseases

Neurodegenerative diseases are a group of disorders originating from the degeneration of central neurons that gradually leads to cognitive and/or motor dysfunctions [74]. These disorders include Alzheimer's disease (AD), Parkinson's disease (PD), Huntington's disease (HD), multiple sclerosis (MS), and Machado-Joseph disease (MJD). With an increase in lifespan, the prevalence of neurodegenerative diseases is increasing significantly [75]. To date, there are no effective treatments for neurodegenerative diseases and the available clinical therapies mainly help in keeping patients from getting worse for a limited period of time [76, 77].

In recent years, the effects of NPY on neurodegenerative diseases and the involved mechanisms have been revealed gradually. Just as reported previously, NPY inhibited the phagocytosis, inflammatory factor release, and motility of activated microglial cells, thereby attenuating microglia-mediated inflammation in injury area of the CNS [78–80]. Aveleira et al. demonstrated that NPY stimulated autophagy of neural cells in in vitro and in vivo experiments, indicating that NPY could promote the clearance of misfolded and abnormal proteins that caused neurodegenerative diseases through autophagy [81]. Excitotoxicity and alteration in calcium homeostasis are related to neurodegenerative diseases. Some studies suggested that NPY reduced glutamate-mediated excitotoxicity and regulated calcium homeostasis [82–85]. Besides, NPY displays some ameliorative effects on clinical manifestations of neurodegenerative diseases. Researchers have investigated the antidepressive effects of NPY in rodent forced swim test, and the results revealed that NPY exerted antidepressant-like properties via Y1 receptor [86–88]. As an orexigenic agent, NPY may ameliorate the weight loss in neurodegenerative diseases [89].

The main physiopathological feature of neurodegenerative diseases is progressive degeneration of neurons; hence, neuronal replacement is considered as a promising therapeutic strategy for neurodegenerative diseases [90]. Recently, an increasing number of studies have demonstrated the regulatory effects of NPY on neuronal precursor cells. NPY facilitates the proliferation of hippocampal precursor cells [32, 33] via Y1-receptor-mediated intracellular NO/cGMP/PKG/ERK1/2 pathway [35]. NPY also promoted the proliferation and neuronal differentiation of SVZ neural precursors [39, 42], as well as the migration of newly formed neuroblast toward striatum and olfactory bulb [41]. The hippocampus and SVZ are the most active areas of neurogenesis in adult brain, and the stimulative effects of NPY on neural precursor proliferation and differentiation in these regions are likely to ameliorate neuron loss in neurodegenerative diseases. Decressac et al. administrated a single ICV injection of NPY to R6/2 mice (a transgenic model of Huntington's disease) and found that NPY improved motor function and extended survival time of the R6/2 mice through reduced body weight loss, meanwhile, NPY promoted cell proliferation and neuroblasts generation in the SVZ of R6/2 mice [91]. In a hippocampal neurodegeneration rat model induced by trimethyltin (TMT), ICV injection of NPY decreased TMT-induced hippocampal damage and stimulated hippocampal neurogenesis through the upregulation of BDNF, Bcl2l1, Bcl-2, Sox-2, Noggin, NeuroD1, and doublecortin genes [92], and the increased newly born neurons later integrated into the local hippocampal circuits [93]. Researchers gave APP-transgenic (tg) mouse (a mouse model of Alzheimer's disease) CNS-targeted delivery of NPY, and they discovered that NPY treatment ameliorated behavioral deficits as well as neurodegenerative pathology of APP-tg mouse and promoted the proliferation of neural precursor cells in subgranular zone of the APP-tg mouse [94].

### 3.2. Retinal Degenerative Diseases

Retinal degenerative diseases, such as age-related macular degeneration, retinopathies, and glaucoma, are a group of ocular diseases characterized by chronic neuronal loss. Currently, there is no cure for retinal degenerative diseases, the main causes of vision loss and blindness. The therapeutic potential of NPY in retinal degenerative diseases has been appreciated due to its neuroprotection and proliferative effect on retinal neural progenitor cells.

It has been shown that exogenous NPY inhibited 3,4-methylenedioxymethamphetamine- (MDMA-) induced necrosis and apoptosis in rat retinal neural cell cultures [95]. Meanwhile, NPY has protective effects against glutamate and N-methyl-D-aspartic acid- (NMDA-) induced rat retinal cell death [83, 96]. NPY could inhibit intracellular calcium concentration ([Ca^2+^]_i_) changes in cultured rat retinal neurons through Y1,Y4, and Y5 receptors [97], which may be one of the mechanisms for the neuroprotective effect of NPY. Furthermore, NPY could inhibit osmotic swelling of Müller cells via Y1 receptor [98, 99], and this glial volume regulatory effect of NPY may be related to its retinal neuroprotection.

Stem cell-based therapy is a novel and promising treatment strategy for retinal degenerative diseases [100]. As mentioned above, NPY promotes the proliferation of retinal neural progenitor cells in primary rat retinal cell cultures [53]. Müller glial cells were identified as a candidate for endogenous retinal stem cells which could differentiate into certain retinal cell types [101–103]. NPY exerts biphasic modulatory effect on proliferation of Müller glial cells. It reduced the proliferation of Müller cells at low concentration, while at higher concentration, it promoted proliferation [104]. Thus, the proliferative effects of NPY on retinal stem/progenitor cells indicate that NPY is a putative target for retina regeneration.

### 3.3. Myocardial Infarction

Stem cell therapy is a promising approach for myocardial infarction (MI) via replenishing cell loss and supplying cytokines that induce angiogenesis or activate resident cardiac stem cell migration and commitment to cardiomyocytes [105]. Some major challenges still plague the stem cell therapy for MI, such as low cell survival rate and poor engraftment of the transplanted cells; therefore, new strategies are desired for better therapeutic effects in stem cell transplantation [106]. The multiple effects of NPY on stem cells, cardiomyocytes, and angiogenesis may be useful in stem cell-based therapy for MI.

It has been shown that NPY pretreatment upregulated the expression of VEGF and mitosis-related genes of rat BMSCs and promoted the migration, endothelium differentiation, and tube formation of BMSCs [55]. Researchers transplanted NPY-pretreated or NPY-untreated BMSCs into the ischemic ventricular wall of myocardial infarcted rats. Four weeks later, they uncovered that NPY pretreatment induced BMSCs to differentiate into cardiomyocytes and endothelium cells in the infarcted myocardium and myocardial infarcted rats which received NPY-pretreated BMSCs exhibited improved cardiac function and reduced ventricular remodeling and fibrosis than those which received untreated BMSCs [55].

In a swine model of chronic myocardial ischemia, NPY treatment improved left ventricular function and enhanced angiogenesis and arteriogenesis via stimulating proangiogenic receptor, growth factor expression, and decreasing antiangiogenic protein expression [107]. In follow-up studies, researchers applying a swine model of hypercholesterolemia and chronic myocardial ischemia demonstrated that local infiltration of NPY in ischemic myocardial improved collateral vessel formation, blood flow, and myocardial function [108], as well as decreased cardiomyocytes apoptosis and myocardium fibrosis in the ischemic region [109]. Furthermore, in vitro experiments revealed that NPY directly induced neonatal and adult rat cardiomyocytes cell cycle reentry and promoted their mitosis and cytokinesis [55]. Thus, given the regulatory effects of NPY on stem cell and the multifaceted beneficial effects on ischemic myocardium, it can be speculated that a combined treatment of stem cells with NPY may be a novel strategy for ischemic heart disease including myocardial infarction.

### 3.4. Osteoporosis

In recent years, the regulatory effects of NPY on osteogenesis and the involved mechanisms have been revealed, which suggested that NPY could be a novel therapeutic target for osteoporosis. Lee and colleagues reported that NPY system inhibited osteoblastic differentiation of mesenchymal progenitor cells and mineralization of mature osteoblasts via the Y1 receptor [58]. Researchers gave C57/BL mice oral administration of BIBO3304 (a selective Y1 receptor antagonist) for 8 weeks and found that this Y1 receptor antagonist increased bone mass of mice in a dose-dependent manner and without adverse extraskeletal side effects [110]. Besides, Liu et al. performing experiments with rat primary BMSCs discovered that NPY stimulated osteoblastic differentiation of BMSCs via activating Wnt signaling pathway [60]. In addition, the promotion of NPY on human BMSC osteogenic differentiation has been demonstrated in in vitro experiments [61]. Although it has not been reported, we can speculate that the regulation of NPY on the osteogenic differentiation of BMSCs may play a role in the treatment of osteoporosis. As recent studies showed, NPY also affected bone homeostasis through regulating hematopoietic stem/progenitor cell (HSPC) mobilization. Park and colleagues demonstrated that NPY induced HSPC mobilization by decreasing HSPC maintenance factor expression via activating matrix metalloproteinase-9 in osteoblasts [63]. And in a mouse model of ovariectomy-induced osteoporosis, NPY treatment ameliorated bone loss by decreasing the number of osteoclasts via increased HSPC mobilization [63]. In subsequent study, this group constructed two NPY-based peptides which were recombined from the cleavage of NPY. These new NPY peptides reduced bone loss in ovariectomized mice more effectively than the full-length NPY, suggesting their potential application in osteoporosis [111].

Furthermore, NPY system still regulates bone homeostasis at the CNS level. In wild-type mice, overexpression of hypothalamus NPY resulted in decrease of osteoblast activity [112]. When Y2 receptor was hypothalamus specific deleted in mice, there was a marked increase in the cancellous and cortical bone volume [113]. The y6R in suprachiasmatic nucleus is required for maintenance of bone mass in mice as it stimulates bone formation and suppresses bone resorption [114]. In general, NPY system exerts multiple regulatory effects on bone homeostasis at CNS and peripheral levels, suggesting that regulating NPY activity may be a novel strategy for osteoporosis treatment.

### 3.5. Obesity

NPY has been demonstrated to have critical effects on the development of obesity through promoting appetite and decreasing energy expenditure [115–117]. Additionally, NPY could directly regulate adipogenesis in peripheral tissues. NPY stimulates the proliferation of hADSCs at low concentration and promotes its adipogenic differentiation at high concentration [69]. The ability of mouse bone marrow stromal cells to differentiate into adipocytes was enhanced in the absence of Y1 receptor [58]. NPY also facilitates the proliferation of rat primary preadipocytes and 3T3-L1 preadipocytes in a concentration-dependent manner via Y1 receptor-mediated activation of ERK1/2 signaling pathway [70] and facilitates adipogenic differentiation of 3T3-L1 preadipocytes via Y2 receptor [71]. In vivo, administration of a NPY pellet in subcutaneous abdominal fat of mice increased the weight and volume of adipose tissue, illustrating the ability of NPY to locally stimulate adipogenesis [71, 118]. Therefore, therapeutic strategies which target the NPY system in peripheral tissue may facilitate intervention for obesity.

## 4. Conclusions

**Figure 2:**
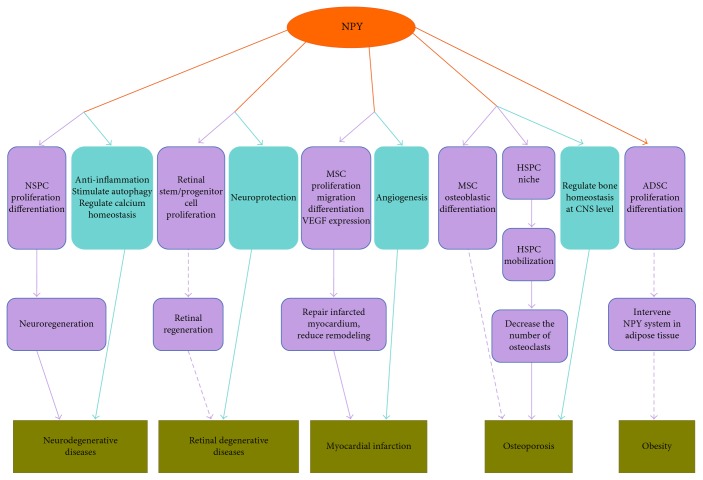
Potential application of NPY's regulatory effects on stem cells. Animal experiments have demonstrated the potential to utilize the regulatory effects of NPY on stem cells for disease treatment (solid violet arrow). Meanwhile, some potential applications could be hypothesized (dotted violet arrow). Besides, given the beneficial effects of NPY itself on these diseases (solid green arrow), combined use of stem cells with NPY may be a novel treatment strategy. NPY—neuropeptide Y; NSPC—neural stem/precursor cell; MSC—mesenchymal stem cell; HSPC—hematopoietic stem/progenitor cells; ADSC—adipose-derived stem cell; CNS—central nervous system.

In summary, NPY exerts multiple regulatory effects on stem cell activities including proliferation, differentiation, self-renewal, factor secretion, migration, and mobilization. NPY could be applied as an adjunct for stem cell grafting and facilitate tissue regeneration as appropriate. Existing data provide evidence that there are potential applications of NPY's regulatory effects on stem cells in treatment of some important diseases (Figure 2). However, it is still in its infancy. The knowledge about effects of NPY on ESCs, HSPC, and induced pluripotent stem cells (iPSCs) are still limited. Further studies are needed to unravel the effects and mechanisms of NPY on different kinds of stem cells, as well as the efficiency and safety in the stem cell-based therapy.
